# Characterisation Data of common pharmaceutical excipient Powders and Tablets for Formulation Development

**DOI:** 10.1038/s41597-026-07707-0

**Published:** 2026-07-01

**Authors:** Dingeman L. H. van der Haven, Maria Mikoroni, René Jensen, Frederik H. Ørtoft, Jan L. Andreasen, Mia E. Andersen, Daniel Dax, Umair Zafar, Andrew Megarry, Kaisa Naelapää, Ioannis S. Fragkopoulos, James A. Elliott

**Affiliations:** 1 University of Cambridge, Department of Materials Science & Metallurgy, 27 Charles Babbage Road, Cambridge, CB3 0FS United Kingdom; 2 Novo Nordisk A/S, Oral Drug Process Development, Måløv, Denmark; 3 Novo Nordisk A/S, Oral Formulation Research, Måløv, Denmark; 4 Novo Nordisk A/S, Devices and Delivery Solutions, Hillerød, Denmark; 5 Novo Nordisk A/S, Finished Product Manufacturing Science and Technology, Hillerød, Denmark

## Abstract

Excipients are essential components within pharmaceutical formulations that are used to enhance properties such as the binding, disintegration, preservation, and patient friendliness of common dosage forms such as tablets, capsules, and inhalers. Common excipients such as micro-crystalline cellulose (MCC), dibasic calcium phosphate dihydrate (DCPD), and partially pre-gelatinised corn starch (PGS) therefore play a critical role in the design and production process of both new and existing drug formulations. However, despite the importance of these powders, reference data are not publicly available. This report presents an extensive data set on MCC, DCPD, and PGS which includes detailed information on individual grains, including particle sizes, crushing, and geometries. Using a compaction simulator, the powders and various mixtures thereof were compressed to make tablets, which were later subjected to a wide range of mechanical tests. This data set provides a reference point for the pharmaceutical industry, educators, and process developers alike.

## Background & Summary

Aside from the active pharmaceutical ingredients (APIs), medicines contain one or more excipients to enhance pharmaceutical effectiveness or manufacturing properties^1,2^. Although the API typically differs between drugs, common excipients are used in many different formulations and many different dosage forms^3^. For example, cellulose can be found in tablets and capsules in various formulations with purposes ranging from pain relief to anti-inflammation and cardiovascular action^4,5^. Moreover, common excipient powders are also widely used in other industries such as agriculture^6^, cosmetics^7^, and food^8^. A detailed understanding of the properties of common excipients is therefore beneficial to the effectiveness and manufacturability of drug formulations. This is especially true for tablets produced using direct compression, since the integrity of a tablet strongly depends on the powder formulation, and challenges are often encountered during scale-up^9,10^.

This report presents an extensive data set on the geometrical and mechanical properties of the following three excipient powders and their binary and ternary blends: micro-crystalline cellulose (MCC), dibasic calcium phosphate dihydrate (DCPD), and partially pre-gelatinised corn starch (PGS). These three powders were chosen from some of the most common excipients for direct compression. Moreover, these powders and their particular grades each have distinct properties that can be considered to be on opposite ends of the spectrum for excipient mechanics^11^. Although lactose is equally common, it was excluded because of scope limitations and overall being less mechanically distinct from the other three excipients considered. The presented powders and their mixtures therefore form good representatives of real pharmaceutical powder formulations^12^. As a result, these powders are not only used in real formulations but also frequently used for placebos, educational purposes, and the development of new formulation techniques and process equipment^13^. For example, the majority of studies on tablet compression use a grade of MCC. Nonetheless, to the best of the authors’ knowledge, a publicly accessible data set of these powders’ compaction profiles is not available.

The data set is a collection of the unpublished data of four studies as well as new data^14–18^. The data set includes measurements on the individual powder grains as well as measurements on the tablets produced from the various powder formulations. The aim of this report is to create a common reference point while improving transparency in industry and academia alike.

## Methods

### Lab environmental control

Novo Nordisk labs, where the work was carried out, employ a rigorous, multi-parameter environmental control strategy underpinned by thermodynamic, fluid dynamic, and acoustic engineering principles. According to EU Pharmacopoeia standards^19^, lab temperatures were maintained between 20 and 25 °C with a relative humidity of 40% or less. Ventilation is engineered on mass-balance and dilution principles, with laboratory air change rates of ≥5/h (typically > 10/h), strictly without recirculation to prevent cross-contamination of aerosols, volatile organic compounds (VOCs), and biological agents. Differential pressure regimes between adjacent zones are maintained through Variable Air Volume (VAV) systems driven by fast-response fume hood automation along with directional airflow. Acoustic climate is controlled by maintaining background noise within 40–45 dB(A), a level scientifically established to provide adequate speech masking in open-plan environments, while requiring a total sound absorption coefficient of A > 0.9 × floor area to limit reverberation. Photometric conditions are standardised at 300 lux for general illuminance and 500 lux at benchtop task level, consistent with the EN 12464-1 laboratory lighting standard. Electromagnetic interference (EMI) is mitigated through galvanically isolated “quiet supply” transformers and shielded cabling in zones housing sensitive analytical instrumentation, reducing conducted and radiated noise that could otherwise compromise measurement accuracy and signal-to-noise ratios. The Building Management System (BMS) integrates all these parameters into a centralised supervisory control and data acquisition (SCADA) framework, enabling real-time monitoring of airflow, pressure differentials, thermal loads, and energy consumption, while allowing systematic reduction of ventilation and heating in accordance with energy optimisation principles.

### Materials

**Fig. 1:**
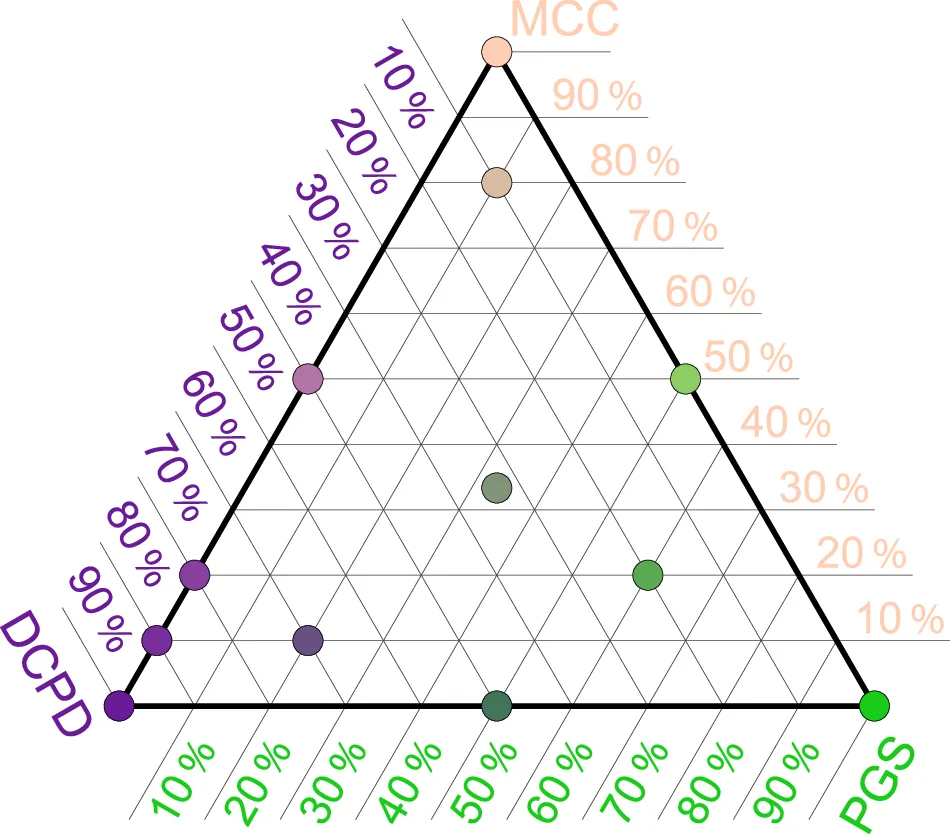
Ternary phase diagram showing the compositions of all the powder formulations presented in this report. The weight percentages of each powder are indicated and always exact multiples of 10. The only exception is the central mixture, whose weight is divided in three equal parts MCC, DCPD, and PGS.

Three pure powders and nine unique mixtures thereof are included in the data set (Fig. 1). The pure powders are: micro-crystalline cellulose (MCC, Avicel PH200^®^, FMC Corporation, Philadelphia, PA, United States), dibasic calcium phosphate dihydrate (DCPD, Emcompress premium^®^, Rettenmaier, Holestebro, Denmark), and partially (pre-)gelatinized starch (PGS, Sepistab^TM^ ST 200, Colorcon, Kent, United Kingdom). The lubricant used was magnesium stearate (MgSt, Peter Greven, Bad Münstereifel, Germany), the most common lubricant for tablet compaction. MgSt was characterised to have a specific surface area of 6 to 10 m^2^ g^−1^ as determined by a TriStar II (Micromeritics Instrument Corp) and using the single-point Brunauer-Emmett-Teller (BET) method. The median particle size by volume ranged from 7 to 11 μm as measured by laser diffraction. The solid state form of MgSt was determined to be a mixture of the monohydrate and dihydrate form, following a combination of differential scanning calorimetry (DSC) analysis (TA instruments, New Castle, Delaware, United States) with a heating cycle from 15 to 250 °C at 2 °C min^−1^ and thermal gravimetric analysis (TGA) measurements (Pyris 1 TGA, Perkin Elmer, Waltham, MA, United States) with a temperature range from 40 to 250 °C at 5 °C min^−1^ ^20^. Both external and internal lubrication methods were used. In the case of external lubrication, an external device (Medelpharm lubrication pack) was used to spray lubricant onto the tooling surfaces before each compaction cycle. In the case of internal lubrication, 1 wt% of MgSt was added to the powder formulation. Blends were created using a 3D shaker TURBULA^®^ T 2 F for 5 min at 25 rounds per minute. All powders and blends were always stored in completely airtight, sealed bags.

All grades were chosen to have similar particle sizes to avoid segregation and exclude changes in formulation properties due to differences in particle size. The chosen grade of MCC has favourable tabletting properties and is considered to be a plastic material (limited potential for particle breakage) with an intermediate stiffness^21–24^. The chosen grade of DCPD has relatively poor tabletting properties for an excipient and is considered to be a brittle material (high tendency for particle breakage) with a high stiffness^21– 23,25^. The hard, crystalline characteristics of DCPD are similar to those of many active pharmaceutical ingredient (APIs), making it a useful mechanical substitute for a general API. The chosen grade of PGS is another frequently used excipient in tablets but is considered to be an elastic or even viscoelastic material (no or very limited particle breakage) with a very low stiffness^22,24,25^. Since these powder grades can be considered to be at the extreme ends of the mechanical properties encountered for excipients, these powders and their mixtures cover a wide range of mechanical properties encountered for tabletting formulations. The compositions of the mixtures were chosen to cover the entire ternary phase diagram with a bias towards DCPD, since DCPD was the most challenging (and therefore most interesting) excipient to compact. Since all of these powders have been studied extensively, they also provide a suitable reference point.

### Laser diffraction

Laser diffraction (LD) was performed using a Mastersizer 3000 (Malvern Panalytical, Malvern, United Kingdom) and the built-in software (version 3.81) was used to obtain a particle size distribution (PSD) through Mie and Fraunhofer scattering theory. All pure powders were measured one to three times, showing negligible variability between measurements. Dry air was used to disperse the powders and assumed to have a refractive index of 1.00. Since the powders contain particles smaller than 50 μm in diameter, optical properties are required. However, these are often unknown and difficult to measure for particulate materials. The powders were therefore all assumed to have a refractive index of 1.54 and an absorption coefficient of 0.00. These present reasonable estimates for most pharmaceutical powders. Moreover, if the particles are larger than 10 μm, small changes in the optical properties have a negligible effect on the resulting PSD^26–29^.

### Optical microscopy

Optical microscopy (OM) was performed using an Olympus BX50 confocal microscope (Olympus Corporation, Tokyo, Japan) equipped with an Euromex camera and either an Olympus UMPlanFl 5× or LCPlanFl 20× objective. For each pure powder, a small amount was deposited on a microscopy slide, which was repeatedly tapped to separate the particles, and then illuminated from below to acquire the images. Per powder, about 2 × 10^3^ to 5 × 10^5^ particles were captured from 113 to 123 images.

### Sieve analysis

Sieve analysis (SA) was performed by hand using six different sieves with woven meshes, placing the coarsest mesh on top with increasingly finer meshes below it. The square openings of these meshes had dimensions of 63 × 63, 90 × 90, 125 × 125, 180 × 180, 250 × 250, and 355 × 355 μm, respectively. Sieving by hand is generally considered to be the reference method in these cases, since mechanical sieving has the potential to break the fragile pharmaceutical powder particles^30^. About 30 grams of each powder was used.

### X-ray computed microtomography

X-ray micro-computed tomography (μCT) was performed using a Zeiss Metrotom 800 (ZEISS, Oberkochen, Germany). The imaging specifications were: 100 kV tube voltage, 40 μA tube current, averaging of 2 frames, 2300 projections, median imaging filter, 6.3 μm voxel size, and Feldkamp reconstruction algorithm (METROTOM software, OS 3.2.4.17214). For each pure powder, a small amount of powder (tens of mg) was suspended in petroleum jelly, also known as Vaseline^®^ (CAS 8009-03-8, Ph. Eur. analytical grade, Merck, Rahway, United States), and pushed into a polyethylene tube with a diameter of 6 mm. More detail on the sample preparation can be found in van der Haven *et al*.^17^. Two samples were scanned per powder, each sample giving a 3150 × 5506 × 5506 μm region for analysis, resulting in about 5 × 10^3^ to 1.0 × 10^5^ particles for each powder. The scanning time per sample was around 1.5 hours.

### Scanning electron microscopy

Scanning electron microscopy (SEM) was performed using a FEI Nova NanoSEM 450 (FEI Company, Hillsboro, Oregon, United States). Secondary electron (SE) images of all pure powders were obtained after dispersing them onto a standard conductive SEM sample holder with carbon tape and coating them with palladium.

### Particle crushing

Single-particle or grain crushing was performed using the Texture Analyser TA.XT.plusC (Stable Micro Systems, Surrey, United Kingdom) with a 5 kg load cell. Each pure powder was sieved as described previously, and the grains were crushed keeping the measurements of each size class separated. Grains were placed in the middle under a flat-faced probe. The probe was set to have a speed of 0.02 mm/s and a trigger force of 0.5 g to start the measurement. The height at the start of the measurement as well as the force and position data were recorded.

### Powder compression

Tablets were made through the direct compression of the powder formulations using a Medelpharm STYL’One Evolution press (Medelpharm, Beynost, France). This is a compaction simulator, a machine that can mimic the processing conditions of the different rotary presses that are usually used to manufacture tablets at a larger scale. The tablet production process is also referred to as tablet compaction or tabletting.

Circular, flat-faced punches with a diameter of *D* = 11.28 mm were used, equipped with displacement sensors (precision of 1 μm) and dual-scale piezoelectric force sensors (precision of 1 N). Powders were always compressed to a targeted tablet thickness of 4.0 mm. However, deviations of up to 0.10 mm are possible due to (delayed) elastic recovery but are usually 0.04 mm or less. The punches had a tolerance gap with the die of 10 μm. The built-in Analis software (AnalisMX v2.08.5, Medelpharm, Beynost, France) was used to correct for the deformation of the punches during compression. The sampling frequency of all sensors was set to 5 or 10 kHz and is clearly indicated by the measurement time stamps in each file.

To be able to measure the radial pressures, the compaction simulator was equipped with a 80 kN load cell or instrumented die^31–33^. The strain gauge within the instrumented die is always 6 mm below the top exit of the die and was calibrated by the manufacturer using 1 to 8 mm gauges of natural rubber (to a precision greater than 0.5%). Punch positions were adjusted such that the centre of the powder bed or tablet was always located at the same height as the strain gauge. Numerical analysis of the die wall with FEM confirmed that the strain fields are very uniform and the measurements remain reliable as long as the former holds true (see supplementary of^16^). More details regarding the tooling, including schematics, can be found in^16,18^.

The standard compaction cycle within this report is a V-shape protocol with constant, linear punch velocities and consists of the following: 1) Filling. The upper punch fully retracts and the lower punch moves down from the top of the die until the desired filling depth has been reached. A force feeder then injects the powder into the die and the excess powder is scraped off from the top of the die. 2) Compression. The upper punch moves down until the strain gauge is exactly in the middle of the two punches. Then, both punches move with equal and opposite velocities towards the centre of the powder bed to compress it into a tablet. 3) Decompression. Both punches move away from the centre of the tablet again at equal and opposite velocities. The upper punch loses contact with the tablet, as does the lower punch, unless the freedom of movement between the lower punch and the actuator allows that punch to move. However, the forces on the lower punch always drop to 0 ± 5 N at the end of decompression. 4) Ejection. The lower punch moves upwards to push the tablet out of the die. To avoid excessive ejection forces due to friction between the tablet and the die, MgSt was added as lubricant (see “Materials” section for details). It will be stated whether internal or external lubrication is used. Finally, the produced tablets are weighed and measured with a microbalance and callipers, respectively. The produced tablets were always stored in completely airtight sealed bags. Any deviations from this protocol will be noted.

#### Single cycle

The standard compression cycle was used to create tablets of all powder formulations, using external lubrication. Each unique powder formulation was compressed to 6 different final densities with 10 to 12 replicates each. The punch speed was 0.2 mm s^−1^, giving a total compression speed of 0.4 mm s^−1^. Generally, these low compression speeds are assumed to give quasi-static conditions, but this is not strictly true^34^. The ejection speed was approximately 300 mm s^−1^.

#### Double cycle

A double compression cycle was used to create tablets of the pure powders, the 50-50 binary mixtures, and the central 33-33-33 mixture. Each of these powder formulations was compressed to 6 different final densities with 3 replicates each. These experiments are exactly the same as the single cycle experiments, except that before ejection the tablet is compressed a second time using the same settings. This type of experiment has been proposed to study the elastic behaviour during constrained compression, while minimising the influence of plasticity by staying below the first cycle’s maximum compaction pressure^35^.

#### Ejection speed variations

Experiments with varying ejection speeds were performed. The standard, single compression cycle protocol was the same for all tests. Only the pure powders with internal lubrication were considered. The punch speed during compression and decompression was 2.0 mm s^−1^, giving a total compression speed of 4.0 mm s^−1^. A total of 5 different ejection speeds were chosen, equally spaced between 0.3 and 280.0 mm s^−1^ on a logarithmic scale. For each ejection speed, each of the pure powders was compressed to 6 different final densities with 3 to 5 replicates each.

A few additional experiments were conducted. The experiments with the PGS powder at the ejection speed 43 mm s^−1^ were repeated with a 125 ms hold time (or dwell time), defined as pause between the end of compression and start of decompression. The experiments with PGS powder were also repeated with external lubrication. Finally, experiments were done with an empty die (no powder) to verify that there is indeed a clearance between the punch and the die and that ejection forces are due to tablet-die friction and not due to the machinery.

#### Effective true density

Following the method by Sun^36^, each powder formulation was compacted to a high pressure. External lubrication was used. The instrumented die was replaced by a regular die (6.00 or 11.28 mm) to withstand the higher radial pressures. The maximum densities and pressures during these experiments can used to determine the effective true density (i.e. the density at a porosity of zero), see “Processed data” section.

### Tablet crushing

Crushing of the tablets was performed using the Texture Analyser TA.XT.plusC (Stable Micro Systems, Surrey, United Kingdom) with a 50 kg load cell. These experiments provide information on the (shear) strength of the tablets, for example as given by the cohesion and internal friction coefficient. From a materials science perspective, Eqs. (1), (2), and (3) are only approximations for the failure stress, but often sufficiently accurate, and we refer to the literature for guidance on their interpretation^18,37–40^.

#### Diametrical compression

Diametrical crushing of the tablets (also known as the Brazilian disk test) was done by placing the tablets on their side and crushing them from above with a circular punch. The maximum force measured then corresponds to the maximum diametrical force before fracture. The diametrical fracture stress can then be estimated assuming a Hertzian contact stress and the Rankine fracture criterion^41,42^, giving

1 $${\sigma }_{{\rm{D}}}^{{\rm{f}}}=\frac{2{F}_{{\rm{D}}}^{\max }}{\pi Dt}.$$

Here, ${F}_{{\rm{D}}}^{\max }$ is the maximum force in the diametrical direction, *t* is the tablet thickness, and *D* is the tablet diameter. Eq. (1) is the official standard for determining the tensile strength of tablets^43^.

#### Axial compression

Axial crushing of the tablets was done by placing the tablets on one of their faces and crushing them from above with a circular punch. The maximum force measured then corresponds to the maximum axial force before fracture. Since the axial tablet strength surpasses the maximum capacity of the Texture Analyser, the compaction simulator described in the “Powder compression” section is used instead. Crushing experiments with the compaction simulator are done by removing the inner die, placing the tablet with the flat side on the lower punch, and then applying a load by moving the upper punch only. This allows the tablet to expand freely, as in crushing with the Texture Analyser. The axial fracture stress can then be estimated, again assuming a Rankine fracture criterion^41,42^, giving

2 $${\sigma }_{{\rm{z}}}^{{\rm{f}}}=\frac{4{F}_{{\rm{z}}}^{\max }}{\pi {D}^{2}}.$$

Here, ${F}_{{\rm{z}}}^{\max }$ is the maximum force in the axial direction, and the other variables are the same as for Eq. (1).

#### Three-point bending

Three-point bending experiments were performed by placing the tablets on two plate-like pillars with a spacing of *L* = 9.75 mm between them. Then, the tablets were loaded at the middle until failure, recording the full force-displacement curves. The three-point fracture stress can then be estimated, again assuming a Rankine fracture criterion^41,42^, giving

3 $${\sigma }_{{\rm{T}}}^{{\rm{f}}}=\frac{3{F}_{{\rm{T}}}^{\max }L}{2{t}^{2}D}.$$

Here, ${F}_{{\rm{T}}}^{\max }$ is the maximum force applied by the loading beam, and the other variables are the same as for Eq. (1). Eq. (3) has been shown to be valid for the flexure of round, flat-faced tablets and has optimal accuracy for the tablets and test setup in this study, having ratios *t*/*D* ≈ 0.35 and *D*/*L* ≈ 1.16^44^.

### Processed data

This section describes a collection of processed data or material parameters that can be derived from the raw data presented in the earlier sections of this report.

#### Particle sizes and shapes

Using the data from laser diffraction, optical microscopy, sieve analysis, and X-ray tomography, the particle size distribution (PSD) and particle shapes were determined. The confidence interval (CI) of the PSD was determined using a bootstrap Monte Carlo method. Particle shapes are reported as either 2D or 3D binary voxel images. Detailed procedures can be found in^17^.

#### Effective true density

The effective true density, or density at a porosity of zero, of each formulation was determined by fitting the Kuentz-Leuenberger model (also known as the modified Heckel model)^45^

4 $$\sigma =\frac{1}{C}\left(\frac{{\rho }_{{\rm{c}}}-{\rho }_{{\rm{m}}}}{{\rho }_{{\rm{t}}}}-(1-{\rho }_{{\rm{c}}})ln\left(\frac{1-\frac{{\rho }_{{\rm{m}}}}{{\rho }_{{\rm{t}}}}}{1-\frac{{\rho }_{{\rm{c}}}}{{\rho }_{{\rm{t}}}}}\right)\right)$$

to the maximum compaction pressures *σ* and compaction densities *ρ*_m_. The resulting fitting constants *C* and *ρ*_c_ are plasticity constants whereas *ρ*_t_ is the effective true density of the material. Since the compaction density *ρ*_m_ refers to in-die as opposed to out-of-die measurements, this leads to a higher effective true density. The original method by Sun, using out-of-die measurement, showed that this method gives lower true densities for water-containing powders when compared to helium pycnometry^36^. Users should keep these differences in mind when interpreting the data. Further details can be found in^14,15^.

#### Ryshkewitch-Duckworth constants

To capture the density dependence, the diametrical and axial fracture stresses were fitted to the Ryshkewitch-Duckworth equation, which is given by^46–48^

5 $${\sigma }^{{\rm{f}}}(\rho )=\bar{\sigma }\exp \left(-{k}_{{\rm{b}}}(1-\rho )\right),$$

with $\bar{\sigma }$ being the fracture stress at *ρ* = 1, *k*_*b*_ being a bonding constant, and the values of *ρ* were determined from the out-of-die tablet density divided by the effective true density. Constant *k*_*b*_ generally follows the trend in tablet composition. More details can be found in^14,15^.

#### Density-dependent Drucker-Prager Cap (dDPC) model parameters

The constitutive model parameters of every powder formulation were determined from the data of the single compaction, effective true density, and crushing (both diametrical and axial) experiments. This includes the density-dependent elastic parameters, being the Young’s modulus and Poisson’s ratio, as well as the plastic parameters for the Drucker-Prager Cap model. Further details can be found in^14,15^.

#### Friction coefficients

For the tablets made out of the pure powders, the following were determined as a function of the tablet density and ejection speed: the ejection force ${F}_{{\rm{ej}}}^{\max }$, the static friction coefficient *μ*_s_, and the kinetic friction coefficient *μ*_k_. All of these refer to the interaction between the tablet and the die wall of the compaction simulator. Further details can be found in^16^.

## Data Records

The full data set is available on Zenodo: https://doi.org/10.5281/zenodo.16732963^49^. Due to the differences in the acquisition equipment and associated software, the extensions of the raw data files are .csv (comma separated text file), .tab (tab separated text file), .xlsx (Microsoft Excel file), .png (portable network graphics), .tif (tagged image file format) or .pdf (portable document format). The processed data are in the same formats, except for the particle size distributions and shapes, which have the extension .mat (MATLAB variable). Each folder contains a README file explaining the contents. Table 1 provides an overview of the top-level data archives and their contents.

**Table 1:** Overview of top-level data archives in the repository. Scale: P = particle-scale; T = tablet-scale; D = derived/processed.

Archive	Scale	Blend	Format	Notes
Light scattering	P	Pure	CSV / XLSX	Particle size distributions by laser diffraction
Optical microscopy – MCC	P	MCC only	PNG	Brightfield micrographs
Optical microscopy – DCPD	P	DCPD only	PNG	Brightfield micrographs
Optical microscopy – PGS	P	PGS only	PNG	Brightfield micrographs
Particle crushing	P	Pure	TAB / XLSX	Single-particle failure loads, force-displacement curves
Scanning electron microscopy	P	Pure	TIF	High-resolution surface morphology images
Sieving	P	Pure	XLSX	Mass-based particle size distributions by sieve fraction
X-ray computed microtomography – MCC	P	MCC only	TIF	3D volumetric greyscale image stack
X-ray computed microtomography – DCPD	P	DCPD only	TIF	3D volumetric greyscale image stack
X-ray computed microtomography – PGS	P	PGS only	TIF	3D volumetric greyscale image stack
Compaction (single) – pure	T	Pure	CSV / XLSX	Single-cycle compression
Compaction (single) – binary	T	Binary	CSV / XLSX	Single-cycle compression
Compaction (single) – ternary	T	Ternary	CSV / XLSX	Single-cycle compression
Compaction (double)	T	All	CSV / XLSX	Double-cycle compression
Compaction (true density)	T	All	CSV / XLSX	Smaller die; effective true density estimation
Compaction (var eject speed)	T	Pure	CSV / XLSX	Varying ejection speeds and lubrication conditions
Tablet crushing	T	All	CSV / XLSX	Axial crushing, diametrical crushing, and three-point bending
Processed data	D	All	XLSX / CSV / TAB / MAT	Derived quantities: particle size and shape statistics, effective true densities, Ryshkewitch–Duckworth constants, density-dependent Drucker–Prager (dDPC) model parameters, and friction coefficients

Blend: “Pure” refers to the three neat excipients (MCC, DCPD, PGS); “Binary” and “Ternary” refer to two- and three-component blends; “All” includes pure powders and all formulations.

File names always contain the exact formulation used. For example, “MCC-DCPD-SEP 10-70-20” indicates a blend of 10 wt% MCC, 70 wt% DCPD, and 20 wt% PGS. PGS (material descriptor) and SEP (trade name abbreviation) were used interchangeably in file names. Tablet data are separated by relative density in separate folders, labelled from “RD0” up to “RD6” by increasing relative density. Similarly, replicate experiments were labelled with “No1”, “No2”, etc. These numbers are consistent across the single-cycle compression data and the diametrical and axial crushing data, thereby indicating the same tablets. The same applies to the data with variations in ejection speed. Measured tablet weights and dimensions are in the .xlsx files within the same compaction and crushing data folders. Particle data files are named by material only (e.g., “MCC”, “DCPD”, or “PGS”), as no replicate or compression-state labelling is required. All raw data files have descriptive column headers including SI units, and image files are accompanied by a separate image with a scale or metadata file with the pixel size.

## Technical Validation

The data presented in sections “Laser diffraction” through “Scanning election microscopy” and in section “Particle sizes and shapes” have been extensively discussed and validated in^17^. The data presented in sections “Single cycle”, “Effective true density”, “Diametrical compression”, “Axial compression”, and from “Effective true density” through “dDPC model parameters” within “Processed data” have been extensively discussed and validated in^14,15^. The data presented in sections “Ejection speed variations” and “Friction coefficients” have been extensively discussed and validated in^16^. The data reported in the remaining sections (“Particle crushing”, “Double cycle”, and “Three-point bending”) have not been discussed previously but were generated using the same validated equipment and general operating procedures.

All data presented in this report were produced from the exact same batches of MCC, DCPD, and PGS. Variations due to changes between individual batches or material grades, the latter of which are known to affect compaction and tablet properties^50^, have therefore been minimised.

**Fig. 2:**
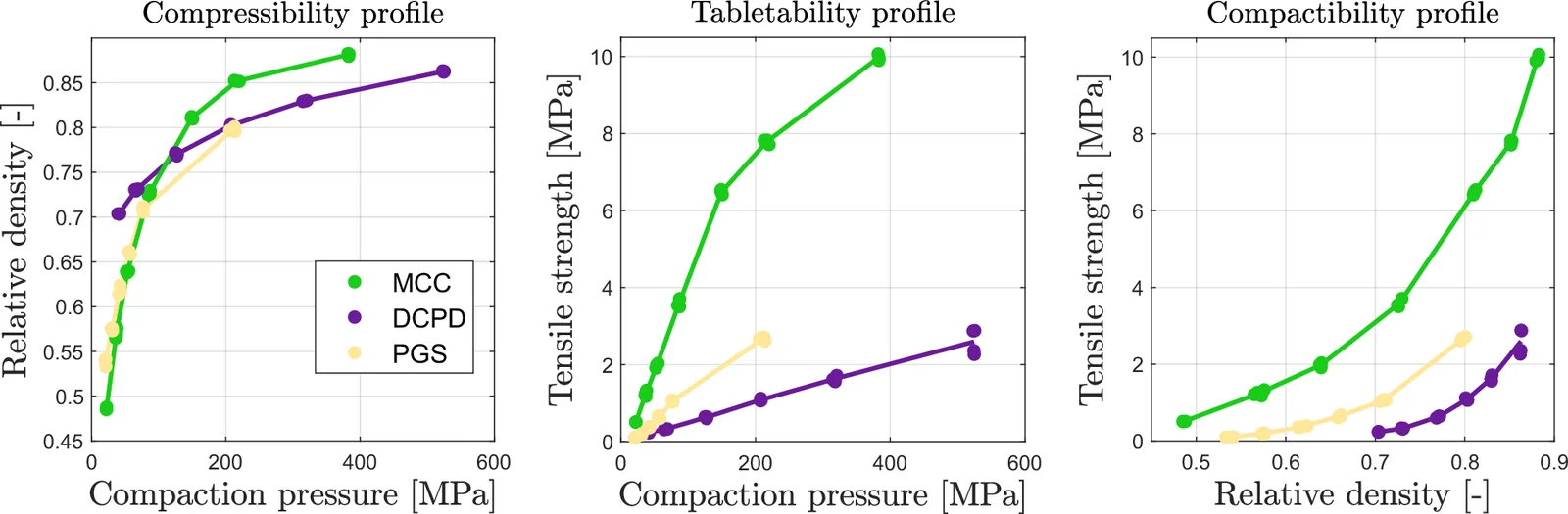
The compressibility, tabletability, and compactibility of the three pure powders.

Figure 2 shows the compressibility, tabletability, and compactability profiles for the three pure powders from the data in sections “Single cycle”, “Effective true density”, and “Diametrical compression”. These are the most critical properties, aside form disintegration time, to asses the suitability for a formulation for tablet production. The behaviour of the powders is as expected from the literature^11,51^. The Sepistab^TM^ variant of PGS is known to result in higher tensile strengths than other starches^52^.

## Usage Notes

The tablet-scale data (compaction and crushing) can be used to derive and develop continuum-based constitutive models^14^. Such constitutive models can then be combined with the finite element method (FEM) to investigate e.g. the effect of tablet shape to identify high stress regions at risk of failure^53,54^. This data set can provide a starting point for researchers before deciding to generate their own data.

The original motivation for the collection of the data presented in this report was to develop a series of mixing rules or methodologies to be able to predict the tablet processing and mechanical properties for an unseen powder formulation. An example of such an approach has been presented in^14,15^.

The particle-scale data (shapes and crushing) can be used for the calibration of a discrete element method (DEM) model, after which the tablet-scale data can be used for validation, such as in^55^. Similarly, the data can be used to develop DEM models for predicting and assessing porosity, flowability, and the efficiency of die filling^56^.

Finally, this data set provides a reference point for pharmaceutical formulation scientists and process developers looking to benchmark powder and tablet properties as well as new numerical methods and equipment.

## Data Availability

The full data set is hosted publicly on Zenodo: https://doi.org/10.5281/zenodo.16732963^49^.

## Code availability

No custom code was used to generate the data. The processing of all data reported in the section “Processed data” was performed using custom in-house MATLAB R2022b scripts developed at Novo Nordisk. The underlying algorithms and workflows have been described in full in the following refs. ^14–17^. These scripts cannot be accessed outside of Novo Nordisk. Further detail may be provided upon request.

## References

[1] Pifferi, G. & Restani, P. The safety of pharmaceutical excipients. *Il Farmaco* **58**, 541–550, 10.1016/S0014-827X(03)00079-X (2003). 12875883

[2] Pockle, R. D., Masareddy, R. S., Patil, A. S. & Patil, P. D. A Comprehensive Review on Pharmaceutical Excipients. *Ther. Delivery*10.4155/tde-2023-0026 (2023). 37464784

[3] Rowe, R. C. *et al*. *Handbook of pharmaceutical excipients*. http://repo.upertis.ac.id/1899/1/Handbook (Pharmaceutical press, 2020).

[4] Akseli, I., Stecuła, A., He, X. & Ladyzhynsky, N. Quantitative Correlation of the Effect of Process Conditions on the Capping Tendencies of Tablet Formulations. *J. Pharm. Sci.* **103**, 1652–1663, 10.1002/jps.23951 (2014). 24668502

[5] Ahmad, U. & Akhtar, J. *Pharmaceutical Formulation Design: Recent Practices*. https://books.google.de/books?id=-pj8DwAAQBAJ (BoD – Books on Demand, 2020).

[6] Anawati, J. & Azimi, G. Recovery and separation of phosphorus as dicalcium phosphate dihydrate for fertilizer and livestock feed additive production from a low-grade phosphate ore. *RSC Adv.* **10**, 38640–38653, 10.1039/D0RA07210A (2020). PMC9057250 35517571

[7] Olivato, J. B. Starch: a natural, safe, and multifunctional ingredient for cosmetic formulations. In *Starch Industries: Processes and Innovative Products in Food and Non-Food Uses*, vol. 3, 255–269, 10.1016/B978-0-323-90842-9.00007-8 (Academic Press, Cambridge, MA, USA, 2024).

[8] Younes, M. *et al*. Re-evaluation of celluloses E 460(i), E 460(ii), E 461, E 462, E 463, E 464, E 465, E 466, E 468 and E 469 as food additives. *EFSA J.* **16**, e05047, 10.2903/j.efsa.2018.5047 (2018). PMC7009359 32625652

[9] Meynard, J., Amado-Becker, F., Tchoreloff, P. & Mazel, V. On the complexity of predicting tablet capping. *Int. J. Pharm.* **623**, 121949, 10.1016/j.ijpharm.2022.121949 (2022). 35752387

[10] Development, Optimization, and Scale-Up of Process Parameters: Tablet Compression. In *Developing Solid Oral Dosage Forms*, 917–951 (Academic Press, London, England, UK, 2017).

[11] Mohammed, H., Briscoe, B. J. & Pitt, K. G. The interrelationship between the compaction behaviour and the mechanical strength of pure pharmaceutical tablets. *Chem. Eng. Sci*. **60**, 3941–3947, 10.1016/j.ces.2005.02.027 (2005).

[12] Jivraj, M., Martini, L. G. & Thomson, C. M. An overview of the different excipients useful for the direct compression of tablets. Pharm. *Sci. Technol. Today* **3**, 58-63 10.1016/S1461-5347(99)00237-0 (2000). 10664574

[13] Zakowiecki, D. *et al*. Exploiting synergistic effects of brittle and plastic excipients in directly compressible formulations of sitagliptin phosphate and sitagliptin hydrochloride. *Pharm. Dev. Technol.* **27**, 702–713, 10.1080/10837450.2022.2107013 (2022). 35913021

[14] van der Haven, D. L. H., Ørtoft, F. H., Naelapää, K., Fragkopoulos, I. S. & Elliott, J. A. Predictive modelling of powder compaction for binary mixtures using the finite element method. *Powder Technol.* **403**, 117381, 10.1016/j.powtec.2022.117381 (2022).

[15] van der Haven, D. L. H., Mikoroni, M., Megarry, A., Fragkopoulos, I. S. & Elliott, J. A. Multi-component mixing and demixing model for predictive finite element modelling of pharmaceutical powder compaction. *Adv. Powder Technol.* **35**, 104513, 10.1016/j.apt.2024.104513 (2024).

[16] van der Haven, D. L. H. *et al*. Tablet ejection: A systematic comparison between force, static friction, and kinetic friction. *Int. J. Pharm.* **661**, 124369, 10.1016/j.ijpharm.2024.124369 (2024). 38914354

[17] van der Haven, D. L. H., Andreasen, J. L., Zafar, U., Fragkopoulos, I. S. & Elliott, J. A. Single-particle geometries of pharmaceutical powders from X-ray tomography; a simple and reliable sample preparation method. *Tomography of Materials and Structures* **8**, 100067, 10.1016/j.tmater.2025.100067 (2025).

[18] van der Haven, D. L. On the compaction of granular matter; continuum and discrete numerical modelling. 10.17863/CAM.113359 (2024).

[19] European Pharmacopoeia (Ph. Eur.) 11th Edition https://www.edqm.eu/en/european-pharmacopoeia-ph.-eur.-11th-edition. [Online; accessed 05. Aug. 2025] (2023).

[20] Wang, T., Potts, A. R. & Hoag, S. W. Elucidating the Variability of Magnesium Stearate and the Correlations With Its Spectroscopic Features. *J. Pharm. Sci.* **108**, 1569–1580 (2019). 10.1016/j.xphs.2018.11.041 30528196

[21] Narayan, P. & Hancock, B. C. The relationship between the particle properties, mechanical behavior, and surface roughness of some pharmaceutical excipient compacts. *Mater. Sci. Eng., A* **355**, 24–36, 10.1016/S0921-5093(03)00059-5 (2003).

[22] Abdel-Hamid, S. & Betz, G. Study of radial die-wall pressure changes during pharmaceutical powder compaction. *Drug Dev. Ind. Pharm.* **37**, 387–395, 10.3109/03639045.2010.513985 (2011). 21446827

[23] Wünsch, I. *et al*. How can single particle compression and nanoindentation contribute to the understanding of pharmaceutical powder compression? *Eur. J. Pharm. Biopharm.* **165**, 203–218, 10.1016/j.ejpb.2021.05.009 (2021). 34010689

[24] Schönfeld, B. V., Westedt, U. & Wagner, K. G. Compression Modulus and Apparent Density of Polymeric Excipients during Compression—Impact on Tabletability. *Pharmaceutics* **14**, 913, 10.3390/pharmaceutics14050913 (2022). PMC9147214 35631499

[25] Patel, S., Kaushal, A. M. & Bansal, A. K. Compression Physics in the Formulation Development of Tablets. *CRT* **23**, 10.1615/CritRevTherDrugCarrierSyst.v23.i1.10 (2006). 16749898

[26] Sperazza, M., Moore, J. N. & Hendrix, M. S. High-Resolution Particle Size Analysis of Naturally Occurring Very Fine-Grained Sediment Through Laser Diffractometry. *J. Sediment. Res.* **74**, 736–743, 10.1306/031104740736 (2004).

[27] Özer, M., Orhan, M. & IşıK, N. S. Effect of particle optical properties on size distribution of soils obtained by laser diffraction. *Environ. Eng. Geosci.* **16**, 163–173, 10.2113/gseegeosci.16.2.163 (2010).

[28] Sochan, A., Polakowski, C. & Łagód, G. Impact of optical indices on particle size distribution of activated sludge measured by laser diffraction method. *Ecol. Chem. Eng. S* **21**, 137–145, 10.2478/eces-2014-0012 (2014).

[29] Bieganowski, A. *et al*. Laser Diffractometry in the Measurements of Soil and Sediment Particle Size Distribution. In *Advances in Agronomy*, vol. 151, 215–279, 10.1016/bs.agron.2018.04.003 (Academic Press, Cambridge, MA, USA, 2018).

[30] Allen, T.*Particle size measurement* (Springer, 2013).

[31] Medelpharm. Die wall pressure measurement system (me-020-en-a) (2010).

[32] Medelpharm. Die wall pressure measurement system (me-019-en-b) (2022).

[33] Medelpharm. Stylcam - instrumented die recommendations for use (me-021-en-a) (2010).

[34] Desbois, L., Tchoreloff, P. & Mazel, V. Use of jump-tests for the characterization of the viscoplastic behavior of pharmaceutical powders during compaction. *Powder Technol.* **404**, 117406 (2022).

[35] Mazel, V., Busignies, V., Diarra, H. & Tchoreloff, P. Measurements of Elastic Moduli of Pharmaceutical Compacts: A New Methodology Using Double Compaction on a Compaction Simulator. *J. Pharm. Sci.* **101**, 2220–2228 (2012). 10.1002/jps.23122 22430162

[36] Sun, C. A novel method for deriving true density of pharmaceutical solids including hydrates and water-containing powders. *J. Pharm. Sci.* **93**, 646–653, 10.1002/jps.10595 (2003). 14762903

[37] Mazel, V. *et al*. Reevaluation of the diametral compression test for tablets using the flattened disc geometry. *Int. J. Pharm.* **513**, 669–677, 10.1016/j.ijpharm.2016.09.088 (2016). 27702696

[38] Mazel, V., Girardot, J., Kopp, J.-B., Morel, S. & Tchoreloff, P. Comparing failure tests on pharmaceutical tablets: Interpretation using experimental results and a numerical approach with cohesive zone models. *Int. J. Pharm.* **642**, 123166, 10.1016/j.ijpharm.2023.123166 (2023). 37356508

[39] Güneyli, H. & Rüşen, T. Effect of length-to-diameter ratio on the unconfined compressive strength of cohesive soil specimens. *Bull. Eng. Geol. Environ.* **75**, 793–806, 10.1007/s10064-015-0835-5 (2016).

[40] Wszola, K.*Modelling and simulation of pharmaceutical tablet failure towards the optimization of powder compaction process*. Master’s thesis, Danish Technical University (DTU) (2023).

[41] Fell, J. T. & Newton, J. M. Determination of Tablet Strength by the Diametral-Compression Test. *J. Pharm. Sci.* **59**, 688–691, 10.1002/jps.2600590523 (1970). 5446428

[42] Goodier, J. N. & Hodge Jr, P. *Elasticity and Plasticity: The Mathematical Theory of Elasticity and The Mathematical Theory of Plasticity* (Courier Dover Publications, 2016).

[43] U.S. Pharmacopea ⟨1217⟩ Tablet Breaking Force https://doi.usp.org/USPNF/USPNF_M99937_02_01.html. [Online; accessed 13. Sep. 2024] (2012).

[44] Mazel, V., Diarra, H., Busignies, V. & Tchoreloff, P. Study of the Validity of the Three-Point Bending Test for Pharmaceutical Round Tablets Using Finite Element Method Modeling. *J. Pharm. Sci.* **103**, 1305–1308 (2014). 10.1002/jps.23898 24523243

[45] Kuentz, M. & Leuenberger, H. Pressure susceptibility of polymer tablets as a critical property: A modified heckel equation. *J. Pharm. Sci.* **88**, 174–179 (1999). 10.1021/js980369a 9950634

[46] Wu, C.-Y., Best, S. M., Bentham, A. C., Hancock, B. C. & Bonfield, W. Predicting the Tensile Strength of Compacted Multi-Component Mixtures of Pharmaceutical Powders. *Pharm. Res.* **23**, 1898–1905, 10.1007/s11095-006-9005-6 (2006). 16850273

[47] Reynolds, G. K., Campbell, J. I. & Roberts, R. J. A compressibility based model for predicting the tensile strength of directly compressed pharmaceutical powder mixtures. *Int. J. Pharm.* **531**, 215–224, 10.1016/j.ijpharm.2017.08.075 (2017). 28823886

[48] Ryshkewitch, E. Compression Strength of Porous Sintered Alumina and Zirconia. *J. Am. Ceram. Soc.* **36**, 65–68, 10.1111/j.1151-2916.1953.tb12837.x (1953).

[49] van der Haven, D. L. H. *et al*. Characterisation data of common pharmaceutical excipient powders and tablets. *Zenodo*10.5281/zenodo.16732963 (2025). PMC13687095 42386792

[50] de la Luz Reus Medina, M. & Kumar, V. Comparative evaluation of powder and tableting properties of low and high degree of polymerization cellulose I and cellulose II excipients. *Int. J. Pharm.* **337**, 202–209, 10.1016/j.ijpharm.2007.01.008 (2007). 17376616

[51] Almaya, A. & Aburub, A. Effect of Particle Size on Compaction of Materials with Different Deformation Mechanisms with and without Lubricants. *AAPS PharmSciTech* **9**, 414–418 (2008). 10.1208/s12249-008-9059-3 PMC2976945 18431664

[52] Perales, M. C. M., Muñoz-Ruiz, A., Antequera, M. V. V., Muñoz, N. M. & Ballesteros, M. R. J.-C. Comparative Tableting and Microstructural Properties of a New Starch for Direct Compression. *Drug Dev. Ind. Pharm*. 10.3109/03639049609063225 (1996).

[53] Kadiri, M. S. & Michrafy, A. The effect of punch’s shape on die compaction of pharmaceutical powders. *Powder Technol.* **239**, 467–477 (2013).

[54] Krok, A., Peciar, M. & Fekete, R. Numerical investigation into the influence of the punch shape on the mechanical behavior of pharmaceutical powders during compaction. *Particuology* **16**, 116–131 (2014).

[55] Zunker, W., Dunatunga, S., Thakur, S., Tang, P. & Kamrin, K. Experimentally validated DEM for large deformation powder compaction: Mechanically-derived contact model and screening of non-physical contacts. *Powder Technol.* **459**, 120972 (2025).

[56] Wu, C.-Y. DEM simulations of die filling during pharmaceutical tabletting. *Particuology* **6**, 412–418 (2008).

